# Rhythm sensitivity in macaque monkeys

**DOI:** 10.3389/fnsys.2013.00049

**Published:** 2013-09-06

**Authors:** Elena Selezneva, Susann Deike, Stanislava Knyazeva, Henning Scheich, André Brechmann, Michael Brosch

**Affiliations:** ^1^Special Lab of Primate Neurobiology, Leibniz Institute for NeurobiologyMagdeburg, Germany; ^2^Special Lab Non-invasive Brain Imaging, Leibniz Institute for NeurobiologyMagdeburg, Germany; ^3^Department Auditory Learning and Speech, Leibniz Institute for NeurobiologyMagdeburg, Germany; ^4^Center for Behavioral Brain SciencesMagdeburg, Germany

**Keywords:** rhythm sensitivity, auditory perception, deviant detection, macaque monkey, auditory cortex

## Abstract

This study provides evidence that monkeys are rhythm sensitive. We composed isochronous tone sequences consisting of repeating triplets of two short tones and one long tone which humans perceive as repeating triplets of two weak and one strong beat. This regular sequence was compared to an irregular sequence with the same number of randomly arranged short and long tones with no such beat structure. To search for indication of rhythm sensitivity we employed an oddball paradigm in which occasional duration deviants were introduced in the sequences. In a pilot study on humans we showed that subjects more easily detected these deviants when they occurred in a regular sequence. In the monkeys we searched for spontaneous behaviors the animals executed concomitant with the deviants. We found that monkeys more frequently exhibited changes of gaze and facial expressions to the deviants when they occurred in the regular sequence compared to the irregular sequence. In addition we recorded neuronal firing and local field potentials from 175 sites of the primary auditory cortex during sequence presentation. We found that both types of neuronal signals differentiated regular from irregular sequences. Both signals were stronger in regular sequences and occurred after the onset of the long tones, i.e., at the position of the strong beat. Local field potential responses were also significantly larger for the durational deviants in regular sequences, yet in a later time window. We speculate that these temporal pattern-selective mechanisms with a focus on strong beats and their deviants underlie the perception of rhythm in the chosen sequences.

## Introduction

Rhythm is considered an ordered recurrent alternation of strong and weak elements in the flow of sound and silence (Merriam-Webster, 2013). The capacity to rhythmically organize isochronous series of external stimuli requires that a subject extracts periodicities from stimuli and makes periodic anticipations of future stimuli. Concomitant with these operations, sensory processing and speed of perception are selectively facilitated for stimuli that are concurrent with the regular temporal grid. This perceptual facilitation is reflected in improved discrimination of pitch (Jones et al., 2002), intensity (Geiser et al., 2012), and temporal cues (Barnes and Jones, 2000; Correa et al., 2006; McAuley and Miller, 2007; Ellis and Jones, 2010; Sanabria et al., 2011; Rohenkohl et al., 2012). Temporal regularities also facilitate auditory stream formation if frequency cues are insufficient, or stabilize streams once they have been formed (Bendixen et al., 2010; Andreou et al., 2011). In addition to perceptual facilitation, rhythm perception may also be reflected in motor behavior; subjects can entrain bodily movements with selected beats of isochronous stimuli (Aschersleben, 2002).

It has long been thought that the ability to synchronize movements to auditory rhythmic beats is unique to humans (McDermott and Hauser, 2005; Fitch, 2009). This has been challenged recently when evidence was presented that several non-human animal species spontaneously moved parts of their body in synchrony with an external stimuli (Patel et al., 2009; Schachner et al., 2009; Cook et al., 2013). These comprised, with the exception of elephants and sea lions, various species of the parrot family only that also show vocal mimicry. Such capacity was neither observed in other birds, like starlings and mynah birds, nor in other mammals like dolphins and non-human primates. The observations of spontaneous rhythmic entrainment were complemented by studies performed under more controlled laboratory situations with operant conditioning procedures in which animals were required, at requested times, to exhibit rhythmic entrainment to receive rewards (Zarco et al., 2009; Hasegawa et al., 2011). Although all three monkeys used in the study of Zarco and colleagues managed to tap reasonably well on a push-button synchronously with periodic auditory and visual stimuli, the animals required excessive training with several hundred thousands of trials, suggesting that rhythmic entrainment is not part of their natural behavior. This is unlike humans in which even 4-year old children with no musical training perform similar tasks easily (McAuley et al., 2006). In addition, rhythmic entrainment of monkeys differed in essential aspects of that of humans. First, the monkeys did not synchronize their tapping with external stimuli with zero phase but had an asynchrony of several hundred milliseconds, suggesting that their taps were responses triggered by the individual stimuli. Second, the monkeys performed similarly well for auditory and visual stimuli or even preferred visual stimuli. This is opposite to humans who show a clear preference for the auditory modality (Patel et al., 2005). It also has to be considered that a test of simply tapping to the pulse of a sequence does not address the question of rhythmic organization on hierarchical levels beyond the pulse of sequential stimuli. Note that the notion that animals are sensitive to rhythms mostly arises from observations of rhythm production. By contrast there is little direct evidence that animals are able to perceive rhythms. We know of no study showing perceptual facilitation in animals.

The studies of rhythm sensitivity in monkeys are of particular interest because of their implications for the evolution of the music and language faculty in humans (McDermott and Hauser, 2005). They also imply that neuronal mechanisms underlying rhythm sensitivity can be studied at the cellular level in an animal model that is closely related to humans. With a few exceptions in animals (Scheich et al., 1979, 1983; Merchant et al., 2011; Perez et al., 2013), such mechanisms have been studied in humans only and with non-invasive brain imaging techniques, like EEG and fMRI. They have revealed the basal ganglia (Matell and Meck, 2004; Buhusi and Meck, 2005; Meck, 2005; Grahn and Brett, 2007; Teki et al., 2011) and several cortical areas including auditory cortex, supplementary motor cortex, anterior cingulate, and premotor cortex (Chen et al., 2008; Coull and Nobre, 2008; Grahn and McAuley, 2009; Grahn and Rowe, 2009; Geiser et al., 2012).

In this report we readdressed the question whether monkeys are rhythm sensitive. Inspired by tests of spontaneous perception, like habituation/discrimination techniques, that have been used in infants (Eimas et al., 1971) and monkeys (Cheney and Seyfarth, 1990) we analyzed changes of facial expressions and gaze spontaneously occurring in monkeys while they were exposed to tone sequences that induce different rhythm percepts in humans. This was supplemented by analyses of neuronal activity recorded from the monkeys' auditory cortex while such sequences were presented.

**Figure 1:**
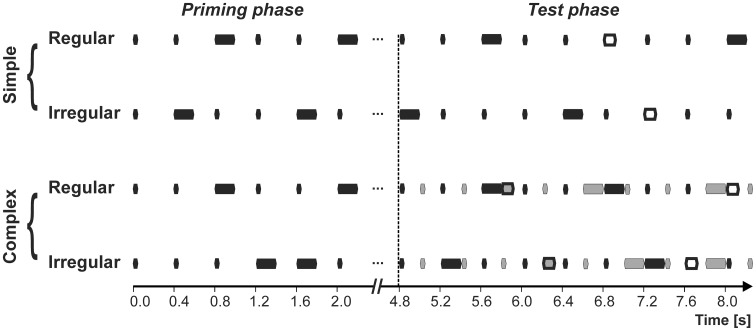
**The four types of tone sequences used in the study.** Black rectangles depict A-tones; gray rectangles depict B-tones. Sequences of different complexity were used, consisting of A-tones only (simple) or of A- and B-tones (complex). Fat-bordered rectangles correspond to duration deviants (white for A-tones, gray for B-tones). A-tones were presented either in an isochronous sequence of repeating triplets of 2 short tones followed by 1 long tone (regular), or in a random isochronous sequence of short and long tones (irregular). The sequence of B-tones was intermittent with the A-tones and was always irregular. The test phase for data analysis was preceded by a priming phase consisting of A-tones only. No deviants were presented during the priming phase.

To study rhythm sensitivity we used isochronous tone sequences (periodic tone onsets) with different patterns of tone duration. The basic sequence was a repeating triplet that consisted of two short tones and a long tone (regular sequence; Figure 1). Human listeners perceptually organize this sequence into triplets with two weak beats followed by a strong beat. To find out whether monkeys similarly organize this tone sequence, we exploited an aspect that is closely associated with rhythm perception, namely perceptual facilitation of stimuli that are concurrent with the regular temporal grid. To this end, we occasionally replaced a long tone in the sequence with a deviant of medium duration and tested whether subjects detected these deviants more easily when they occurred in a regular sequence than they did when the deviants occurred in an irregular sequence of short and long tones (Figure 1). In regular sequences, subjects, in principle, could exactly predict both the timing of future events and their duration, i.e., they were able to form a specific template, relative to which deviance could be detected. For irregular sequences, by contrast, there was uncertainty about the duration of future events such that the template was less well defined and deviance detection was less easy.

For a validation of deviant detection as an indirect measure of rhythm sensitivity, i.e. as a disruption of rhythm, we first performed a psychophysical experiment on human subjects who were instructed to indicate duration deviants by button presses. In this experiment, we also addressed the question whether our deviance detection paradigm is suitable to infer rhythm sensitivity for more complex tone sequences. These complex tone sequences were composed of a simple sequence, either with a regular or an irregular duration pattern, and a distractor sequence of tones of different timbre that was intermittently presented and whose duration pattern was irregular (Figure 1). After completion of this experiment, we exposed monkeys to the same sequences and searched for changes of gaze and facial expressions relative to occurrence of the deviants and for different rates of changes in regular and irregular sequences. This study of spontaneous behavior was complemented by microelectrode recording of neuronal discharges and local field potentials from auditory cortex while monkeys were exposed to the same sequences. Here, we analyzed whether the neuronal responses to the tones of different duration differentiated regular from irregular sequences. In particular, we analyzed whether the differences were strongest for the long tones, i.e., for the tones that form the strong beats in the regular sequences.

## Material and methods

### Stimuli

The stimuli were digitally synthesized (Matlab, The Mathworks Inc., Natick MA) harmonic complex tones comprising 10 partials with a *F*_0_ of 440 Hz, lasting 50 ms, 100 ms and 200 ms, including 5 ms linear onset and offset ramps. These tones were generated with two different amplitude envelopes which resulted in clearly different and easily discriminable timbres. In the A-tones, the amplitudes increased from the first to the tenth partial by 12 dB per partial. In the B-tones, the amplitudes fell in reverse order from the tenth to the second partial by 12 dB per partial. The fundamental component had always the maximum amplitude. For the human subjects, the A- and B-tones were adjusted for equal subjective loudness by increasing the level of the B-tones by 3 dB.

From these tones, four types of isochronous sequences were generated (Figure 1). The two simple sequences consisted of 75 A-tones only (50 short and 25 long), which were presented at a constant inter onset interval (IOI) of 400 ms, yielding a total sequence duration of 30 s. They were presented either in a repeating pattern of two short tones of 50 ms duration each and a long tone of 200 ms duration (regular), or presented in random order (irregular). The 3rd and 4th sequence type were complex, i.e., were a superposition of one of the two simple tone sequences and a distractor tone sequence. The distractor sequence started 4.8 s after sequence onset and consisted of 63 B-tones (42 short and 21 long tones). The B-tones were presented between the A-tones (with an IOI of 200 ms between A and B tones) and varied randomly between being of short (50 ms) or long duration (200 ms).

For the psychophysical and neurophysiological experiments described below, 5 out of the 25 long A-tones (as well as 5 of the 21 long B-tones if present) were substituted, at random positions and at the earliest 4.8 s after sequence onset, by tones of medium duration of 100 ms. These duration deviants were suprathreshold for monkeys (Sinnott et al., 1987). Thus, the initial 4.8 s were free of deviants and served as a “priming phase” to inform the subjects about the temporal patterning of the A-tones. Five different randomizations of deviant positions (sequence exemplars) were created for each of the four sequence types.

### Experiment in human subjects

Twelve listeners (seven male, five female) aged between 20 and 39 years participated in the psychophysical experiment. All subjects had normal audiograms, with absolute thresholds ≤20 dB hearing level. All subjects gave written informed consent to the study, which was approved by the Ethics Committee of the University of Magdeburg.

The psychophysical measurements were performed in an acoustically shielded chamber (IAC). Each of the five exemplars of the four types of tone sequences was presented two times to a subject during an experimental session, resulting in 10 presentations per sequence type. All tone sequences were presented in pseudo-randomized order across the experiment and alternated with a silence interval of 20 s. The stimuli were presented diotically via headphones (Sennheiser, HD 465) at an individually adjusted, comfortable level using Presentation (Neurobehavioral Systems Inc.).

The subjects were instructed to follow the A-tones and to ignore the B-tones, if present, and to indicate duration deviants among the A-tones by pressing the left mouse button with their index finger. Button presses were counted as hits when they occurred in the response window between 300 and 1000 ms after the onset of a duration deviant in the A-sequence. False alarms were all button presses outside this response window. From these measures we calculated the sensitivity index d' (Swets, 1961) for each sequence type and each subject. The sensitivity index d' was then subjected to a repeated-measure ANOVA testing for the within-subject effects of “Regularity” (regular or irregular) and “Complexity” (simple or complex).

### Experiments in monkeys

Experiments were performed on five adult cynomolgus monkeys (*Macaca fascicularis*). All five monkeys participated in the psychophysical experiment (monkeys Ec, Ed, El, We, and Wi) and two of them (monkeys El and We) in the neurophysiological experiment. All monkeys appeared to have normal hearing as indicated by their general auditory behavior. The experiments were approved by the authority for animal care and ethics of the federal state of Saxony Anhalt (No. 43.2-42502/2-802 IfN) and conformed to the rules for animal experimentation of the European Communities Council Directive (86/609/EEC).

Experiments were carried out in a double-walled sound-proof room (IAC 1202-A). Auditory stimuli were generated in a computer, AD converted and amplified. In the psychophysical experiment, identical stimuli were presented simultaneously through two free-field loudspeakers (Canton Karat 720), placed ~1 m and 90 degrees to the left and right of a subject, at a sound pressure level of about 75 dB. For neurophysiological recordings, stimuli were presented diotically through earphones (Etymotic 4S; we did not use free-field loudspeakers because we performed additional tests on the same experimental days that required earphones).

In the psychophysical experiment, the monkeys sat in a primate chair, which permitted them to rotate around their vertical body axis and to move their arms, legs, and lower trunk. Monkeys were videotaped during tone presentation with an Ikegami ICD-848P Digital camera, using Pinnacle Studio software (25 frames per second).

As will be described below, human subjects were better in detecting the duration deviants for simple sequences. Therefore, we initially tested all monkeys with the simple regular and irregular sequences only. On an experimental day we selected two exemplars, one of the five exemplars of regular sequences and another one of the five exemplars of irregular sequences. The first selected exemplar was presented 30 times, with a silence interval of 10 s between presentations. After a pause of ~3 min, this was followed by 30 presentations of the second exemplar. This succession of exemplar presentations was repeated once, either on the same experimental day or on another day. Thus, each monkey was presented with 300 deviants in regular sequences and 300 deviants in irregular sequences. This block design instead of presenting regular and irregular sequences in random order (as in the human study) was used to help the monkeys to perceive the duration pattern. Two monkeys who exhibited spontaneous behavior that was related to the tone sequences were also exposed to complex sequences with regular and irregular patterns.

During a prescreening of the video recordings, we found out that changes of the monkeys' gaze (“looking up”, i.e., vertical eye movements) could very well be used to describe spontaneous reactions of the monkeys to deviants. We also noted that these changes were frequently accompanied by changes of facial expression (i.e., raising eye brows). Therefore, short video clips were cut from the video recordings, each showing the time window of 100–650 ms (14 frames) after the onset of a deviant. An example clip showing a change of a monkey's gaze and facial expression is provided in the supplementary video file (‘Movie 1; note that the frame rate is reduced to 5 frames/s, blue rectangle in the left bottom corner indicates when deviant is present). The clips were scored by three raters for the occurrence of such a behavioral response (“looking up”). The raters were blind to the sequence type because they scored the clips in randomized order and were not able to hear the tones. Clips were considered to reflect a response to a deviant (hit) if at least two of the three raters scored a behavioral response. For the simple sequences, we also analyzed whether such behavioral responses occurred at time windows before deviant presentation. For this purpose, raters scored (in the same way as described above) video clips showing a 550-ms-long time window (14 frames) which started 1.2 s before deviant onset.

In two monkeys (Ec and We), the raters were able to score most of 1800 video clips, except for 183 clips in monkey Ec and 285 clips in monkey We. In the latter clips neither facial nor eye movements could be identified because the camera lost to view the gaze of the monkeys. In three other monkeys (El, Ed, Wi), the raters identified very few (<5) changes of the monkeys' gaze (or other consistent behaviors) in the video clips. Thus, sufficient data for further statistical analysis was available for two monkeys only.

The neurophysiological experiment was performed in one monkey (We) who showed spontaneous behavioral responses to deviants and in another monkey (El) who did not show such behavioral responses. The monkeys were head fixated through a headholder device (see Brosch and Scheich, 2008 for details on headholder implant and other methods). Recordings were made with a multichannel system (Thomas Recordings) which enabled us to move up to five microelectrodes through a skull opening into left (monkey We) or right (monkey El) auditory cortex. Based on the tonotopic gradient and the recording depths, most recordings were estimated to be from the primary auditory cortex. Following preamplification, the signals from each of the electrodes were amplified and filtered (PGMA-64, Thomas Recordings). The filter settings were 1–200 Hz to yield local field potentials and 0.5–5 kHz to yield action potentials of small groups of neurons (multiunits). Local field potentials and action potentials were recorded with an A/D data acquisition system (Neuralynx), with sampling rates of 659 Hz and 42 kHz, respectively. From selected multiunit records, the action potentials of single units were extracted off-line with a template-matching algorithm.

For each multielectrode recording, the four sequence types were presented in the same pseudo-randomized order as in the study on humans but with a silent interval of 10 s between presentations. Each sequence type was presented six times.

For each of the four sequence types and the three durations of A-tones we calculated a post stimulus time histogram (PSTH) from the action potentials of a unit relative to tone onset, with a bin size of 20 ms. To ease comparison across units each PSTH was normalized to the mean firing rate which was obtained from the twenty three 10-s silent intervals between the presentations of sequence exemplars. To control for first-order sequential facilitatory or suppressive effects of the immediately preceding tone (Brosch and Scheich, 2008), only tones following a short tone were included in these analyses. To examine the steady state condition only, we included only tones after the 4.8-s priming phase. Similar to the action potentials, we calculated auditory evoked potentials from the local field potentials, low-pass filtered at 65 Hz. Again, only tones following a short tone after the priming phase were included in these analyses.

## Results

### Human psychophysics

**Figure 2:**
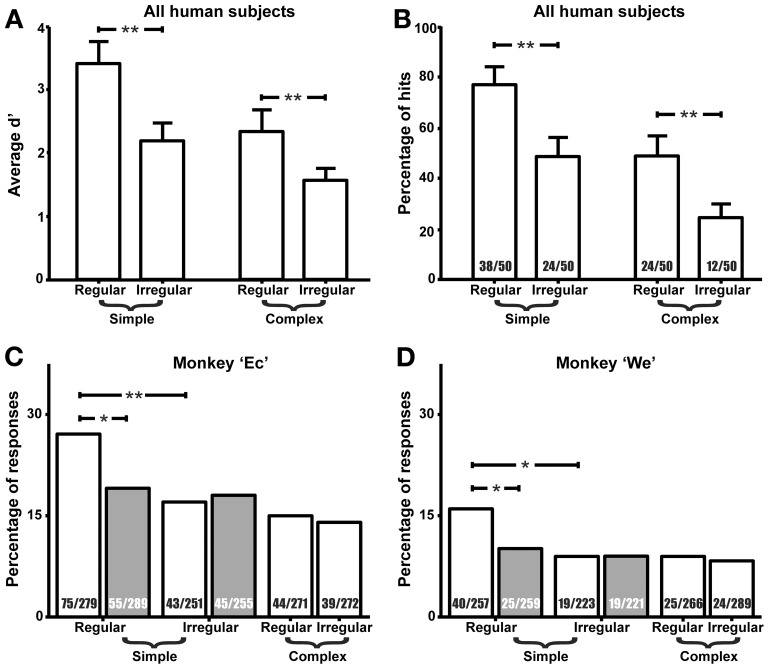
**Regular arrangement of tones can improve the detection of duration deviants in humans and monkeys. (A)** Average sensitivity index d' of twelve human subjects for four sequence types. Hairs show standard errors. Asterisks indicate results of *t*-tests (^**^p < 0.01). **(B)** Average percentage of correct responses to duration deviants (hits) in twelve human subjects. Average number of hits and number of presented deviants are shown within each bar. **(C,D)** Percentage of spontaneous behavioral responses to deviants (empty bars) and in time windows before deviant presentation (filled bars) in two monkeys. In (**B–D)**, asterisks indicate results of chi-square tests (^*^p < 0.05, ^**^p < 0.01). Number of behavioral responses and number of scored video clips (black for deviants, white for non-deviant time windows) are shown within each bar. Note that the human data were obtained in a forced-choice task and the monkey data during spontaneous behavior.

Twelve human subjects were assessed by means of the sensitivity index d' for their ability to detect duration deviants in the four sequence types, which differed in their temporal duration pattern (regular and irregular) and complexity (simple and complex). For these subjects, the repeated-measures ANOVA of sensitivity index d' revealed a significant main effect (Figure 2A) of the within-subject factor “Regularity,” with a higher d' in regular than in irregular sequences [*F*_(1, 11)_ = 26.04, *p* = 0.0003]. Furthermore, the ANOVA revealed a significantly higher d' for simple than for complex sequences {main effect “Complexity,” [*F*_(1, 11)_ = 36.43, *p* = 0.00009]}. The interaction between the factors “Complexity” and “Regularity” did not reach significance (*p* > 0.1).

We also described the human sensitivity in terms of the percentage of correct responses to duration deviants (hits), the results of which are given for all subjects in Figure 2B. This showed that response rates varied strongly between 25% (for complex irregular sequences) and 77% (for simple regular sequences). When the behavior of individual subjects was considered, we found that 9 of the 12 subjects scored significantly more hits for regular than for irregular sequences (Chi-square test, *p* < 0.05), both for simple and complex sequences. Two subjects had significantly different hit rates either only for the simple or for the complex sequences. The remaining subject exhibited hardly any responses to duration deviants regardless of the sequence type.

### Spontaneous behavior of monkeys

In two of the five monkeys we identified spontaneous behavior (“looking up”) that occurred in the time window from 100 to 650 ms after the onset of a duration deviant in the tone sequence. Each of the two monkeys exhibited such behavior significantly more frequently when the deviant occurred in a regular sequence rather than in an irregular sequence, but only for simple sequences (Figures 2C,D; Chi-square test: χ^2^ = 7.26, *p* < 0.01 in monkey Ec; χ^2^ = 5.50, *p* < 0.05 in monkey We) and not for complex sequences (χ^2^ = 0.38 in monkey Ec; χ^2^ = 0.16 in monkey We). For simple sequences the percentage of spontaneous reactions decreased from 27 to 17% in monkey Ec and from 16 to 8% in monkey We. These reactions also occurred significantly more frequently after the deviant onset than in the time window before deviant presentation, but only for simple regular (χ^2^ = 4.96, *p* < 0.05 in monkey Ec; χ^2^ = 4.1, *p* < 0.05 in monkey We) and not for simple irregular sequences (χ^2^ = 0.02 in monkey Ec; χ^2^ = 0.001 in monkey We). In the three other monkeys the percentages of spontaneous reactions were too small to allow for comparisons across the 4 sequence conditions.

### Neuronal activity in auditory cortex of monkeys

**Figure 3:**
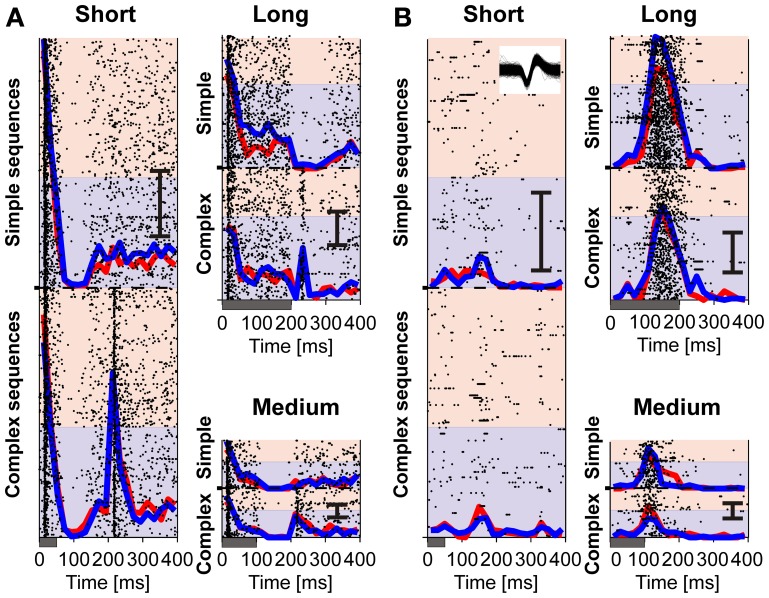
**Two example units in auditory cortex whose firing discriminates simple regular from irregular tone sequences. (A)** Firing of a multiunit for three tone durations (short, long, medium). Responses to the regular sequences are plotted as raster displays on the pale blue background; responses to the irregular sequences are plotted on the pale red background. In the raster display, each dot in a horizontal raster line indicates the occurrence of a spike relative to onset of an A-tone. Within each colored block responses are ordered along the ordinate from bottom to top in sequential order of stimulus presentation. Duration of A-tones is indicated by bars below each panel. Average firing rates (PSTHs) for regular (blue lines) and irregular (red lines) sequences are superimposed. The scale bar represents 30 spikes/s. **(B)** Firing of a single unit. Inset shows all waveforms of its spikes. Note increased firing to long tones only in simple regular sequences (blue lines) in panels **(A)** and **(B)**.

We analyzed 175 units recorded in two monkeys (74 in monkey We and 101 in monkey El) for the influence of the duration pattern of tone sequences on the neuronal firing in auditory cortex. The general observation was that auditory cortex neurons fired more strongly to regular than to irregular sequences. This increase in firing was most pronounced in response to the long A-tones and was found in simple sequences only. These findings are exemplified by the representative multiunit shown in Figure 3A whose firing discriminated simple regular from irregular sequences between 60 and 140 ms after the onset of a long A-tone. Similar observations were made in other multiunits and single units (e.g., Figure 3B).

**Figure 4:**
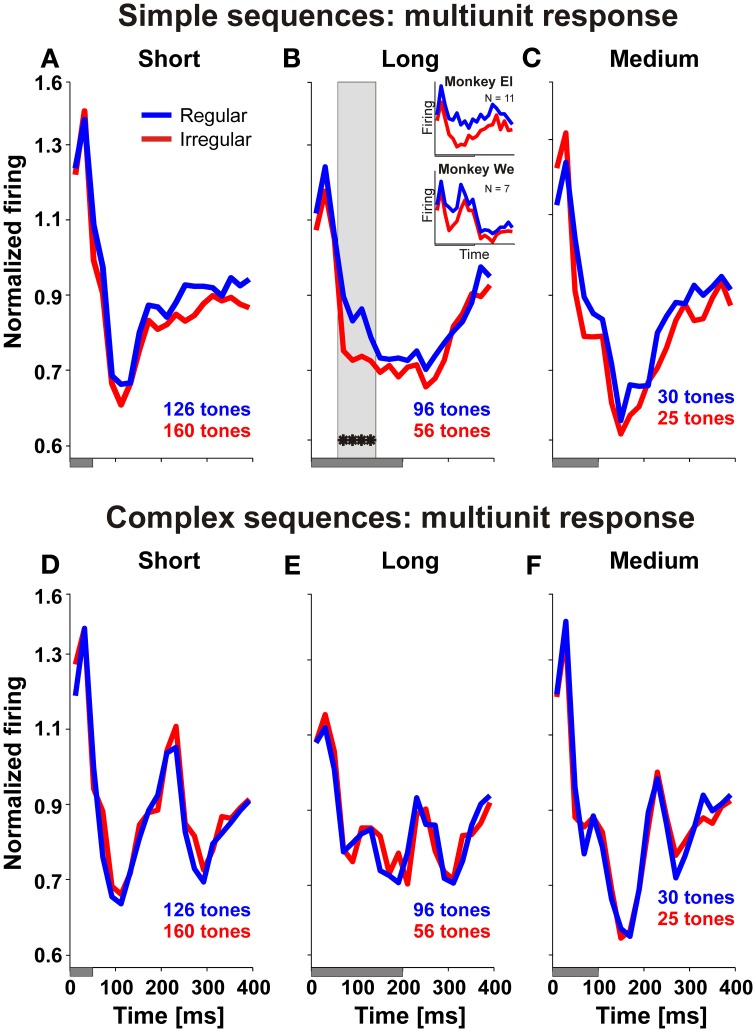
**Neuronal firing in auditory cortex discriminates the temporal structure of tone sequences.** Each of the panels **(A–F)** compares the median response of all 175 units recorded in two monkeys relative to the onset of an A-tone for regular (blue) and irregular (red) sequences. Durations of A-tones are indicated by gray bars below each panel. Significant differences are indicated by asterisks (*p* < 0.01, Wilcoxon signed rank test). Gray rectangle in panel **(B)** show the time window we used for further multiunit analysis which revealed units whose firing was stronger in simple regular sequences than in simple irregular sequences. The median response of these units is shown in the inset of panel **(B)**, separately for two monkeys.

To quantitatively and comprehensively describe the influence of regularity on auditory cortex we firstly compared the median response of the entire sample of 175 units to the tones in regular and irregular sequences, separately for simple and complex sequences (Figure 4). This revealed significantly stronger responses from 60 to 140 ms after onset of the long A-tones only when they were part of simple regular sequences (Figure 4B; we only considered the population responses to be significantly different if two or more consecutive bins were different at *p* < 0.01, Wilcoxon signed rank test). These observations suggest an effect of regularity and complexity of tone sequences on the neuronal firing in auditory cortex that is selective for a specific time window, namely during the long tone. It also suggests that the duration patterning had no sustained effect on neuronal firing.

To examine which of the 175 units exhibited such discriminative responses to the long tones we checked with a *t*-test (*p* < 0.01, one-sided) whether the response of a given unit from 60 to 140 ms after onset of an A-tone was greater when it was presented in a simple regular rather than in a simple irregular sequence. Our analysis revealed that 18 units (10.3%; 7 units in monkey We and 11 units in monkey El), including the units shown in Figure 3, responded more strongly to long A-tones when the sequence was regular and simple. Note that the insets in Figure 4B show very similar responses to the long tones of these units in both monkeys despite their difference in spontaneous behavior. For complex sequences only one unit responded more strongly to the long A-tone in regular sequences.

**Figure 5:**
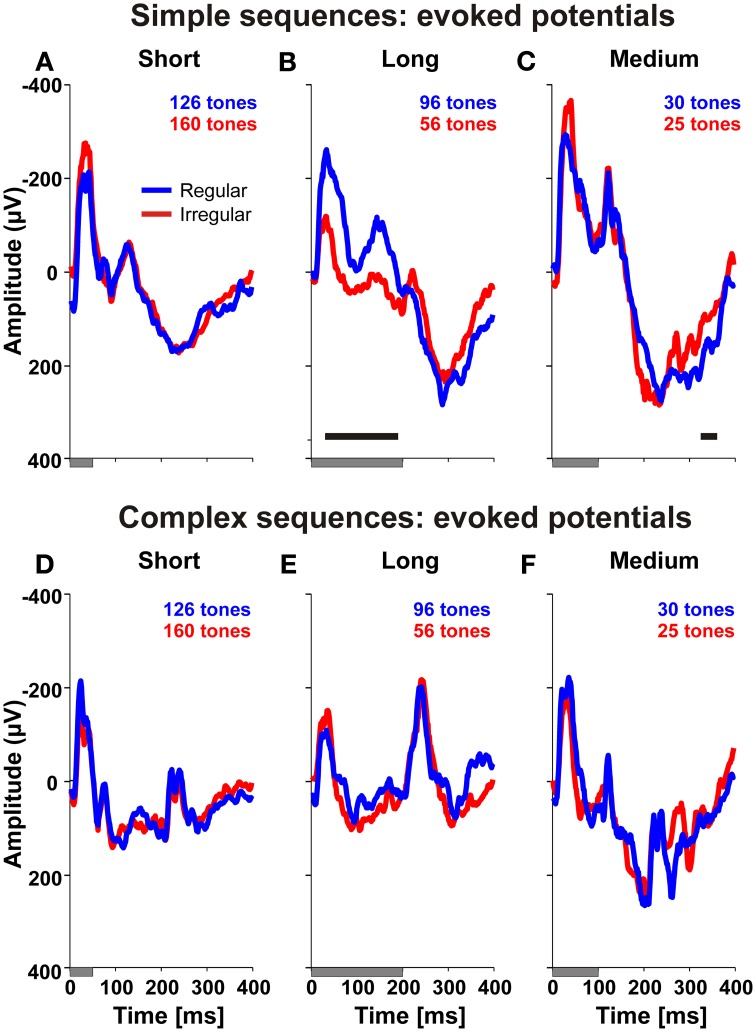
**Local field potentials in auditory cortex discriminate the temporal structure of tone sequences.** Each of the panels **(A–F)** compares the median local field potential determined from 175 recording sites in two monkeys relative to an A-tone for regular (blue) and irregular (red) sequences. Durations of A-tones are indicated by gray bars below each panel. Significant differences are indicated by black bars in the panels (*p* < 0.01, Wilcoxon signed rank test).

A similar analysis performed on local field potentials confirmed that neuronal activity in auditory cortex discriminated regular from irregular sequences. Figure 5 shows the median evoked potentials determined from all 175 recording sites for A-tones of short, medium and long duration for sequences with different regularity and complexity. Significant differences between regular and irregular sequences (*p* < 0.01, to be compatible with the spike analysis, more than 13 consecutive sampling points of the local field potential, corresponding to 20 ms, had to be significantly different at *p* < 0.01, Wilcoxon signed rank test) were found in the time windows of 30–190 ms after onset of a long A-tone when this tone was presented in a simple sequence. During the time window of 30–190 ms, the evoked potential to long A-tones at 9 of the 175 sites discriminated regular from irregular sequences (*p* < 0.01, one-sided *t*-test). In addition, local field potentials had larger excursions in regular sequences in later time windows from 325–360 ms after tone onset but only for the tone deviants of medium duration, presented in simple sequences. This parallels the different spontaneous behavioral response probabilities of the monkeys to the deviant tones in simple regular and irregular sequences.

## Discussion

The current study shows that there are macaque monkeys whose spontaneous behavior and whose neuronal discharges and local field potentials in auditory cortex differentiated isochronous tone sequences with regular temporal patterns from isochoronous sequences with irregular patterns. In the following, we argue that these findings provide evidence that macaque monkeys have the ability to sense the rhythmic organization of tone sequences.

The first evidence is that the observation that two of five monkeys more frequently changed their gaze or facial expressions in response to occasional deviants when they appeared in a sequence of regularly arranged short and long tones. Such deviance detection is often thought of as an automatic brain process that compares a sensory stimulus to a “template” formed by a frequent background stimulus (Näätänen et al., 1978; Fishman and Steinschneider, 2012). For regular sequences, deviance detection may have been promoted because the repeating pattern of triplets of two short and one long tone provided information to predict the duration of future tones, i.e., subjects could form a specific template relative to which a deviance could be detected. For irregular sequences the random duration patterning provided no information to predict the duration of future tones such that the template was less defined and thus deviance detection was not promoted. As a result of increased predictabilities, duration sensitivity was improved. Because of this perceptual facilitation induced by the duration pattern more deviants were detectable in regular sequences, which more frequently attracted the attention in some monkeys and subsequently modified their spontaneous behavior or triggered new behavior. This could also explain why in our psychophysical study on humans, albeit under forced choice conditions, subjects had higher hit rates to duration deviants for regular sequences. The fact that only two of the five monkeys showed the spontaneous behavior is no counterargument to their general ability to perceive deviants as a violation of the triplet structure since no reinforcement was used to evoke any special interest in the deviants.

Our finding that deviance detection was different for regular and irregular sequences suggests that monkeys are able to discriminate the sequential patterning of tone duration, i.e., are able to recognize triplets of two short and one long tone. Our observations may be explained on the basis of grouping, without perception of periodicity of the tone sequences. That is, subjects may hear the regular sequences as repeating groups of short-short-long (with a grouping boundary after the long tone) and then detect the disruption of this pattern. This distinction is important because rhythm perception has at least two subcomponents: grouping (the perceptual segmentation of sound streams into phrases or chunks) and beat/meter (the perception of an underlying periodicity of the isochronous sequence, involving prediction of the timing of beats; Lerdahl and Jackendoff, 1983). Because we only tested isochronous sequences, we cannot provide direct evidence whether our behavioral results are mostly due to the sensitivity to temporal pattern (short-short-long) or to the sensitivity to periodicity. This question has to be addressed in future studies. However, since perceptual facilitation is stronger for isochronous sequences than for anisochronous sequences (Large and Jones, 1999; Jones et al., 2002; Quené and Port, 2005), the behavioral results do not exclude that perception of periodicity of the tone sequences is essential for the increased deviant detection in regular sequences, and that monkeys, like humans, are sensitive to periodicity. Because recognition of the repeating triplet pattern required monitoring a period containing at least three tones (>1.2 s), current findings also support previous reports that monkeys are able to hold information of auditory stimuli in short-term memory for several s (Colombo and D'Amato, 1986; Fritz et al., 2005; Scott et al., 2012).

A more direct support that monkeys may be sensitive to sequence periodicity comes from our analyses of neuronal activity in their auditory cortex. We found neurons whose tone responses differentiated the duration pattern of tone sequences. The differential firing was selective for a specific time window in the tone sequence, closely overlapping the long tones and resulting in an enhancement of the responses to these tones. This was paralleled by similar differences in the tone-evoked field potentials. These findings suggest the existence of a time-selective mechanism in the auditory system operating beyond the pulse of the isochronous sequence (Ulanovsky et al., 2004; Schroeder and Lakatos, 2009), which results in a selective enhancement in the responses to the long tones relative to the responses to the short tones. We speculate that this response contrast enhancement underlies the perceptual organization of the regular tone sequences into repeating triplets of two weak beats and one strong beat. This interpretation is supported by several considerations. There is no obvious reason as to why only the long tones are enhanced by neuronal activity in regular sequences, if the main issue is recognition of triplets by grouping process only. Even if one would argue that the recognition of the long tone at the end of the triplet reflects the occurrence of the complete triplet, the enhancement of response does not seem to be an adequate neuronal correlate in this experimental context. Monkeys were just exposed to the tone sequences, without any task or reinforcement being involved. Under such circumstances, one would expect habituation to the repeating triplets rather than an enhancement of responses. On the other hand, enhancements of neuronal responses to repeating auditory patterns are seen in auditory cortex when predictions of these patterns and of subsequent rewards can be made (Selezneva et al., 2006; Brosch et al., 2011). Therefore, predictability of events seems to be at least one factor which contributes to the enhancement of long tone responses in regular sequences (note that rhythm sensitivity implies a predictive concept). If one considers the unique perceptual role of the long tone as carrier of the strong beat that determines the rhythm across the triplets, this consideration seems plausible to explain the results. This also seems to explain some compatible and some adverse results in the auditory cortex of anesthetized rats (Yaron et al., 2012). In that neuronal study, responses differentiated isochronous tone sequences with regular frequency patterns from those with irregular frequency patterns. Opposite to our results, however, neuronal responses were stronger for irregular patterns. We speculate that this may reflect differences between composition of sequences (frequency patterns vs. durational patterns), state of animals (anesthetized vs. awake), or differences between species (monkeys vs. rats). It should be noted that sequences composed of tones of different frequencies do not necessarily elicit the sensation of different beats.

The human and monkey results are in agreement that regular sequences can be better differentiated from irregular sequences if the sequence is simple (consisting only of one sound type with variable duration, here termed A-tone) rather than complex (consisting of the A-tones and additional sounds with a different timbre, termed B-tones). This suggests that the B-tones distracted the subjects and impeded their ability to extract the duration patterning of the A-tones and to make predictions of future A-tones. The distracting effect of the B-tones may even be more pronounced because these tones (1) had the same pitch and (2) were always presented exactly between the A-tones, creating a common isochronous temporal grid within which A- and B-tones were arranged. The resulting “common fate” of the A- and B-tones may have promoted their perceptual integration into a common auditory stream (Bregman, 1990), within which the duration patterning of the A-tones was masked. While this informational masking may provide an explanation for the degraded duration sensitivity of human subjects, current data about spontaneous behavior and neuronal activity in auditory cortex of monkeys cannot clarify the question how strong is the impairment in this species. The distractors could render the monkeys completely incapable to perceive the duration patterning of the A-tone; or the distractors may be less effective when monkeys are forced by task demands to selectively attend the A-tones, as in the human experiment. The difference between simple and complex sequences is compatible with a recent study that failed to find differences in EEG signals in monkeys that reflected the rhythmic structure when a highly complex sound sequence was presented (Honing et al., 2012).

Evidence for rhythm sensation in monkeys was obtained by observing their spontaneous behavior and the firing of neurons in auditory cortex. This suggests that the cognitive ability to hierarchically organize a stimulus sequence is part of the “natural” and innate behavior of monkeys and is not “artificially created” by excessive behavioral training (Zarco et al., 2009). The fact that only two of the five monkeys showed the increase of spontaneous deviant-related orientation responses in regular sequences is not a counterargument. It is possible that we will observe such behavior also in the remaining three monkeys if they were videotaped for longer periods or in situations different from being in a primate chair. It is also possible that spontaneous interest in the deviants may vary among animals. The fact that the two examined monkeys showed enhancements of neuronal responses to long tones (one in the right and the other in the left auditory cortex) even though one of them did not show the behavioral responses indicates that the neuronal capability to mark the strong beats is rather reliable and not an individual trait.

Consequently our results imply that macaque monkeys are sensitive to the rhythmic structure of stimulus sequences. Furthermore, we suggest that some of the perceptual characteristics of the music and language faculty in humans (McDermott and Hauser, 2005; Fitch, 2009) may be based on brain mechanisms already present in monkeys. Whether the ability to perceive rhythms can be transferred to the motor realm was not addressed in the current study. Our results also suggest that some of the characteristics of the monkeys' rhythm perception and possibly of other cognitive abilities can be tested by analyzing their spontaneous behavior and does not always require excessive behavioral training with reinforcement.

## Conflict of interest statement

The authors declare that the research was conducted in the absence of any commercial or financial relationships that could be construed as a potential conflict of interest.
